# Molecular Mechanisms Underlying Neuroinflammation Intervention with Medicinal Plants: A Critical and Narrative Review of the Current Literature

**DOI:** 10.3390/ph18010133

**Published:** 2025-01-20

**Authors:** Sandra Maria Barbalho, Beatriz Leme Boaro, Jéssica da Silva Camarinha Oliveira, Jiří Patočka, Caroline Barbalho Lamas, Masaru Tanaka, Lucas Fornari Laurindo

**Affiliations:** 1 Department of Biochemistry and Pharmacology, School of Medicine, Universidade de Marília (UNIMAR), Marília 17525-902, São Paulo, Brazil; smbarbalho@gmail.com (S.M.B.); lucasffffor@gmail.com (L.F.L.); 2 Postgraduate Program in Structural and Functional Interactions in Rehabilitation, School of Medicine, Universidade de Marília (UNIMAR), Marília 17525-902, São Paulo, Brazil; 3 Department of Biochemistry and Pharmacology, School of Medicine, Faculdade de Medicina de Marília (FAMEMA), Marília 17519-030, São Paulo, Brazil; 4 Faculty of Health and Social Studies, Institute of Radiology, Toxicology and Civil Protection, University of South Bohemia Ceske Budejovice, 37005 Ceske Budejovice, Czech Republic; 5 Department of Chemistry, Faculty of Science, University of Hradec Kralove, 50003 Hradec Kralove, Czech Republic; 6 Department of Gerontology, School of Gerontology, Universidade Federal de São Carlos (UFSCar), São Carlos 13565-905, São Paulo, Brazil; 7 Danube Neuroscience Research Laboratory, HUN-REN-SZTE Neuroscience Research Group, Hungarian Research Network, University of Szeged (HUN-REN-SZTE), Tisza Lajos Krt. 113, H-6725 Szeged, Hungary

**Keywords:** neuroinflammation, neurodegenerative diseases, medicinal plants, oxidative stress, microglia, nuclear factor kappa B (NF-κB), nuclear factor erythroid 2-related factor 2 (Nrf2), Janus kinase/signal transducer and activator of transcription (JAK/STAT), NLRP3 inflammasome, phytochemicals

## Abstract

Neuroinflammation is a key factor in the progression of neurodegenerative diseases, driven by the dysregulation of molecular pathways and activation of the brain’s immune system, resulting in the release of pro-inflammatory and oxidative molecules. This chronic inflammation is exacerbated by peripheral leukocyte infiltration into the central nervous system. Medicinal plants, with their historical use in traditional medicine, have emerged as promising candidates to mitigate neuroinflammation and offer a sustainable alternative for addressing neurodegenerative conditions in a green healthcare framework. This review evaluates the effects of medicinal plants on neuroinflammation, emphasizing their mechanisms of action, effective dosages, and clinical implications, based on a systematic search of databases such as PubMed, SCOPUS, and Web of Science. The key findings highlight that plants like *Cleistocalyx nervosum* var. paniala, *Curcuma longa*, *Cannabis sativa*, and *Dioscorea nipponica* reduce pro-inflammatory cytokines (TNF-α, IL-6, and IL-1β), inhibit enzymes (COX-2 and iNOS), and activate antioxidant pathways, particularly Nrf2. NF-κB emerged as the primary pro-inflammatory pathway inhibited across studies. While the anti-inflammatory potential of these plants is significant, the variability in dosages and phytochemical compositions limits clinical translation. Here, we highlight that medicinal plants are effective modulators of neuroinflammation, underscoring their therapeutic potential. Future research should focus on animal models, standardized protocols, and safety assessments, integrating advanced methodologies, such as genetic studies and nanotechnology, to enhance their applicability in neurodegenerative disease management.

## 1. Introduction

The increase in life expectancy has been accompanied by a rise in the prevalence of age-related Neurodegenerative Diseases (NDs). Maintaining neuronal function and cognitive ability depends on the brain’s high energy demands, as it is one of the most energetically active organs despite not performing mechanical work like the skeletal muscles or heart [1,2,3,4]. Typically, the human brain consumes approximately 3.5 mL of oxygen per 100 g of brain tissue per minute [5,6,7]. This high oxygen consumption makes neurons susceptible to Reactive Oxygen Species (ROS) damage and subsequent inflammation [8,9,10,11,12,13,14,15,16].

Neuroinflammation is characterized by activating the brain’s innate immune system in response to inflammatory stimuli. This response involves the activation of microglia and astrocytes, the release of pro-inflammatory cytokines and chemokines, ROS production, and, sometimes, the infiltration of peripheral leukocytes into the Central Nervous System (CNS). While neuroinflammation initially acts as a protective mechanism, sustained or maladaptive inflammation has been identified as a critical factor in the progression of various neurological diseases, including NDs, psychiatric disorders, pain syndromes, stroke, and traumatic brain injury [4,17,18,19,20,21,22]. Substantially, neuroinflammation involves the dysregulation of several signaling pathways, including Nuclear Factor Kappa B (NF-κB), Nuclear Factor Erythroid 2-Related Factor 2 (Nrf2), Nucleotide-Binding and Oligomerization Domain-Like Receptor Family Pyrin Domain-Containing 3 (NLRP3), and Janus Kinase (JAK)/Signal Transducer and Activator of Transcription (STAT), at different proportions. Typically, NF-κB, NLRP3, and JAK/STAT are detrimental, while Nrf2 possesses antioxidant and anti-inflammatory effects [23,24,25,26].

Addressing neuroinflammation to reduce disease severity and improve patient outcomes is a promising strategy against neurodegeneration. From a molecular perspective, there are several conventional drug targets for neuroinflammation, such as enzymes, receptors, and ion channels [22,27,28,29,30,31]. However, the high cost of synthetic drugs presents a challenge, emphasizing the need for alternative approaches [32,33,34,35]. This has heightened interest in naturally occurring medicinal plants known for their antioxidant, anti-inflammatory, and neuroprotective properties. These plants are often more cost-effective and have been safely utilized in treatments for thousands of years [36,37,38].

A substantial body of literature underscores the beneficial effects of dietary phytochemicals and medicinal plants on overall health and longevity [39,40,41,42]. Medicinal-plant-based drug discovery remains a vital and underexplored area of research with substantial potential [43,44,45,46]. Despite pharmaceutical advances, plants have historically been a rich source of therapeutic compounds, yet many of their potential benefits are still untapped [47,48]. For example, resveratrol, a phytochemical widely found in grapes, has the potential to modulate inflammation within the CNS effectively by targeting critical pathways involved in microglia polarization and neuronal cell survival, and research advises incorporating resveratrol sources into dietary routines to decrease the risk or delay the onset of NDs [49,50,51,52,53]. By comprehensively investigating these plants, researchers can uncover novel compounds that could offer significant breakthroughs in treating various conditions. Specifically, this approach could lead to the discovery of new treatments for complex issues such as neuroinflammation and neurodegeneration, which are often challenging to address with current therapies [13,46,54,55].

**Figure 1:**
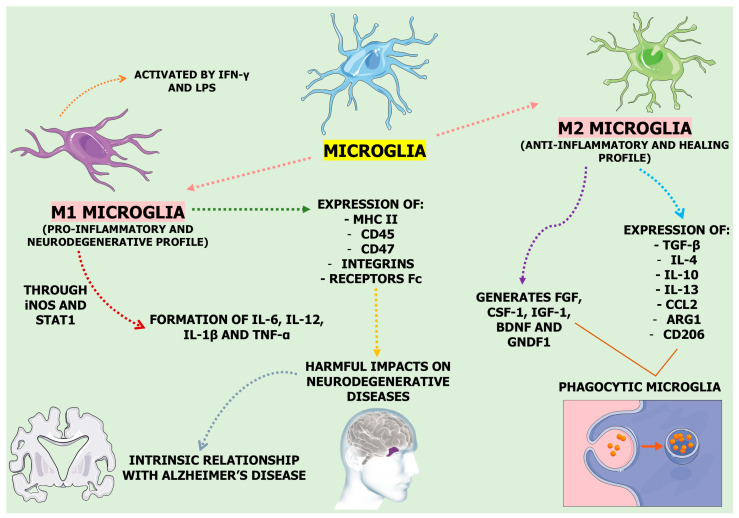
Microglia have M1 and M2 phenotypes depending on conditions. In a homeostatic state, they support neuronal health through synaptic pruning and clearing misfolded proteins. Under stress, they shift to the pro-inflammatory M1 phenotype, releasing Reactive Oxygen Species (ROS) and cytokines that can damage neurons. When exposed to Transforming Growth Factor Beta (TGF-β), interleukin (IL)-4, IL-10, or IL-13, they switch to the anti-inflammatory M2 phenotype, aiding in phagocytosis, Extracellular Matrix (ECM) rebuilding, and neuronal survival. Abbreviations: ARG, Arginase; BDNF, Brain-Derived Neurotrophic Factor; CCL, Chemokine (C-C motif) Ligand; CD, Cluster of Differentiation; CSF, Colony-Stimulating Factor; FGF, Fibroblast Growth Factor; GNDF, Glial cell line Derived Neurotrophic Factor; IGF, Insulin-like Growth Factor; IFN-γ, Interferon Gamma; iNOS, Inducible Nitric Oxide Synthase; LPS, Lipopolysaccharide; MHC-II, Major Histocompatibility Complex Class II; STAT, Signal Transducer and Activator of Transcription; TNF-α, Tumor Necrosis Factor-Alpha.

Despite previous efforts, the existing reviews have yet to fully explore the potential of medicinal plants in regulating neuroinflammation. Tyler et al. [56] offered valuable insights into the therapeutic potential of plants for NDs. However, their review needed a systematic examination of the literature addressing medicinal plants targeting neuroinflammation. Their focus was broader, with a greater emphasis on ethnobotanical aspects rather than exclusively discussing neuroinflammation. In contrast, Suk et al. [57] explored the regulation of neuroinflammation by herbal medicine and its implications for NDs. Their review centered predominantly on traditional medicines and their flavonoid derivatives. Additionally, this review, published in 2005, needs to be updated and would benefit from an update incorporating recent advancements in the field. Due to the limitations of the already published research regarding medicinal plants targeting neuroinflammation, our study aims to not only update the current body of research, integrating cutting-edge recent publications in its findings but also provide a systematic and critical evaluation of how medicinal plants affect signaling pathways and specific molecular targets to modulate neuroinflammation, ultimately giving powerful insights into the molecular aspects of neuroinflammation treatment under green healthcare and advocating for future research endeavors in this field, promoting a future with fewer diseases. This manuscript includes a table (Table 1) that compiles research on medicinal plants’ roles as potential neuroinflammation modulators. Figure 1 illustrates the microglia’s distinct phenotypes, M1 (pro-inflammatory) and M2 (anti-inflammatory).

## 2. Targeting Molecules and Signaling Pathways in Neuroinflammation Intervention

### 2.1. Nuclear Factor Kappa B (NF-κB): A Central Player in the Development of Neuroinflammation and Neurodegenerative Conditions

In 1986, Sen and Baltimore discovered that in B lymphocytes, a protein binds to a specific Deoxyribonucleic Acid (DNA) sequence called NF-κB that will act in case of extensive inflammation [76]. NF-κB has the property of transcribing genes, which may be pro-inflammatory, triggering a series of events related to neuroinflammation [77]. The two catalytic subunits, Inhibitory Kappa B (IκB) Kinase Alpha (IKKα) and IκB Kinase Beta (IKKβ), and the regulatory subunit NF-κB Essential Modulator (NEMO) comprise the IκB Kinase Complex (IKK) [78]. Two different pathways activate NF-κB, so the canonical pathway is primarily responsible for inflammation and is stimulated by Toll-Like Receptors (TLRs). First, IκBα captures the p65/p50 dimers in the cytoplasm. Consequently, certain pro-inflammatory stimuli, such as pathogens or cytokines, release p65/p50 dimers through the phosphorylation cascade so that IκBα is degraded. Soon after, the cognate kB motif is linked to p65/p50 translocated in the nucleus, activating the NF-κB gene [77]. The regulatory protein Myeloid Differentiation Primary Response 88 (MyD88) mediates the canonical process [78]. In this sense, there is a negative feedback mechanism since the temporary activation of NF-κB is related to the induction of negative regulators, such as IκBα, A20, and p105 [79]. The non-canonical NF-κB pathway has RelB/p52 as the transcription factor and maintains an intimate relationship with IKKα homodimers, considering that it depends on them to occur [79]. Interestingly, in certain types of cells, some members of NF-κB, such as RELA, associate with p100 and are activated through the induction of p100 processing [80]. In this context, regulating this pathway is only possible through NF-κB-Inducing Kinase (NIK) expression. Thus, NIK recruitment to Tumor Necrosis Factor Receptor-Associated Factor (TRAF) 2 at a steady state is mediated through TRAF3, resulting in the ubiquitination of NIK and its consequent degradation. Therefore, the NF-κB complex remains inactive in the cytoplasm due to low levels of endogenous NIK. Upon activation of the non-canonical NF-κB pathway, TRAF3 proteolysis is induced by TRAF2. This degradation of TRAF3 releases and accumulates NIK. After this, phosphorylation of p100 by IKKα heterodimers is induced by NIK, promoting an incomplete deterioration that makes p52 available. Ultimately, p52-RelB heterodimers translocate to the nucleus, allowing target genes to be transcribed [81].

Since the host may be exposed to pathogens that cause tissue damage and infections, inflammation is a protective response of the body capable of promoting the degradation of pathogens and wound healing [80]. In the case of neuroinflammation, the CNS promotes an immune response, which is sometimes related to the etiology and course of several psychiatric and NDs, such as Alzheimer’s disease, Parkinson’s disease, and Multiple Sclerosis. In this sense, NF-κB in the glial cells has a central role in the regulation of chronic neuroinflammation, which is neurodegenerative [82,83], bearing in mind that various diseases can be exacerbated or intensified in cases of uncontrolled inflammation [84,85].

### 2.2. Nuclear Factor Erythroid 2-Related Factor 2 (Nrf2): A Novel Approach to Address Oxidative Stress and Neuroinflammation in Neurodegenerative Disorders

It is essential to understand that Nrf2, discovered in 1994 [86], remains low until induced by an oxidative increase inside the cell, resulting in the degradation of this protein and its movement to the nucleus. In principle, Nrf2 is an essential Leucine Zipper Protein (bZIP) that belongs to the Cap ’n’ Collar (CNC) family of transcription factors, which also comprises NRF1, NRF3, and p45 NF-E2 and 605 amino acids [87,88,89]. It is interesting to note that Nrf2 exerts protective action against inflammation by inhibiting NF-κB [90]. While NF-κB is extensively studied for its role in promoting neuroinflammation, its crosstalk with the Nrf2 pathway—a critical regulator of antioxidant responses—remains underexplored in disease-specific contexts. Understanding how these pathways influence each other could unveil novel therapeutic strategies, especially given that Nrf2 activation can suppress NF-κB-driven inflammation through antioxidant mechanisms. Exploring this interplay might be crucial for developing integrated approaches to managing neuroinflammatory conditions. In an oxidative stress scenario, Nrf2 is the most important regulator of phase II genes and binds to Antioxidant Response Elements (AREs). Establishing a Kelch-Like Epichlorohydrin-Related Protein (Keap1)/Nrf2 complex in the cytoplasm makes it possible to affirm that the Nrf2/ARE pathway is organized by the negative regulator Keap1. Nrf2 phosphorylation is induced through Keap1 so that it forms heterodimers (the small musculoaponeurotic fibrosarcoma oncogene homolog Nrf2-Maf) after being translocated to the nucleus [91,92,93]. Some pathways indicate that phosphorylation is occurring, and they are the ones that activate Nfr2: Phosphatidylinositol 3-Kinase (PI3K), Casein Kinase 2 (CK2), Protein Kinase C (PKC), and Mitogen-Activated Protein Kinase (MAPK) [94]. The control of many antioxidant pathways is carried out through Nfr2 target genes. One can mention, as an antioxidant pathway, the synthesis and regeneration of Glutathione (GSH) and the synthesis and recovery of thioredoxin [95].

Given the relevance of Nrf2 for numerous processes in the body, it is crucial to highlight its relationship with neuroinflammation and neurodegeneration. Firstly, it is essential to point out that Nrf2 is expressed in the CNS, which involves cells such as microglia and neurons [96]. Microglial cells play a central role in neuroinflammation, and through two distinct activation pathways, they change from the M0 state (resting state) to the M1 or M2 state (activated state). The M1 state is pro-inflammatory and provides pro-inflammatory cytokines, including Tumor Necrosis Factor-Alpha [TNF-α] and interleukin [IL]-6, increasing the amount of tissue damage through the generation of ROS [84,97,98]. Given this, increased levels of oxidative markers and signaling, in addition to constituent parts of damaged cells, could be seen in patients with diseases such as Parkinson’s disease, Multiple Sclerosis, and Alzheimer’s disease [89,99]. Therefore, activating the Nrf2-ARE pathway induces the action of antioxidant proteins and detoxifying enzymes, which defend the body from inflammatory damage [100]. In this scenario, Nrf2 has activators that can neutralize the injuries caused by ROS that promote neurodegeneration [101].

### 2.3. Impact of the Nucleotide-Binding and Oligomerization Domain-like Receptor Family Pyrin Domain-Containing 3 (NLRP3) Inflammasome on Neuroinflammation: Exploring a Promising Therapeutic Target for Neuroinflammation

There are three mechanisms of NLRP3 activation: canonical, non-canonical, and alternative pathways. However, before canonical activation, the NLRP3 inflammasome must be prepared to control the expression of components present in it, such as NLRP3 itself, pro-IL-1β, and procaspase-1 [102]. In the first stage, which is called the priming stage or signal one and requires the activation of MyD88 [103], specific Pathogen-Associated Molecular Patterns (PAMPs) and Damage-Associated Molecular Patterns (DAMPs) relate to Pattern-Recognition Receptors (PRRs), such as Nucleotide-Binding Domain and Leucine-Rich Repeat Containing Receptors (NLRs), Nucleotide-Binding Oligomerization Domain-Like Receptors (NODs), or TLRs. It promotes an intense induction and transcription of NF-κB, which causes an increase in the expression of the NLRP3 protein. After this, the deubiquitination of NLRP3 and the ubiquitination and phosphorylation of the Apoptosis-Associated Speck-Like Protein (ASC) occur, which allows the assembly of the inflammasome. Furthermore, this step is responsible for inducing the Post-Transductional Modifications (PTMs) of NLRP3, which make NLRP3 stable in a state without activity and self-suppressed but with an appropriate signal [104,105,106,107,108]. Following the activation step, the NLRP3 oligomer, pro-caspase 1, and ASC are prepared to structure a complex during oligomerization [109]. This phase is known as inflammasome assembly or signal 2, and it is activated by certain molecular and cellular stimuli, such as PAMPs, DAMPs, Adenosine Triphosphate (ATP), and ion influx. In this scenario, the pro-inflammatory cytokines IL-18 and IL-1β and the pyroptosis effector protein, Gasdermin-D (GSDMD), are cleaved by the pro-caspase-1 enzyme so that IL-18 and IL-1β are released extracellularly after the N-terminal fragments of GSDMD create pores in the plasma membrane. It is also important to point out that ionic and osmotic flow occurs through these pores, and the influx of calcium (Ca^2+^) and efflux of potassium (K^+^) promote cell turgidity so that the influx of Ca^2+^ releases cellular vesicles and the efflux of K^+^ promotes an intensification of inflammasome activation [110,111,112,113,114,115]. Furthermore, the adaptive immune response is induced by IL-18 and IL-1β [116]. In humans, caspase-4 triggers a non-canonical response, which occurs as a reaction after Gram-negative bacteria infect the organism [117]. Unlike the previous pathways, in the alternative path, Lipopolysaccharide (LPS) stimulation induces the maturation and consequent secretion of IL-1, in addition to the activation of caspase-1. Furthermore, this pathway requires Spleen Tyrosine Kinase (Syk) activity, caspase 4/5, and Ca^2+^ flux influenced by LPS and mediated by the Cluster of Differentiation (CD)14/TLR4 [106].

Given the working mechanism of NLRP3, it is possible to state that it has an intrinsic relationship with neuroinflammation and NDs. The activation of NLRP3 inflammasomes in the CNS is related to misfolded proteins and amyloids where, in pathological situations, these proteins have the role of being a signaling molecule that initiates the transcription and expression of NLRP3 and IL-1β, producing a cascade inflammatory character and positive feedback [118]. Studies have shown that patients with Parkinson’s disease have, in their cerebrospinal fluid, high concentrations of IL-1β and IL-18, in addition to an upregulated gene expression of caspase-1, ACS, and NLRP3, as well as increased protein expression of IL-1β, caspase-1, and NLRP3 in cells with only one nucleus in peripheral blood [119,120]. In this sense, the activation of microglial inflammasomes stimulated by Alpha-Synuclein (α-SYN) and TLR is related to the progression of Parkinson’s disease [121,122]. Alzheimer’s disease is also associated with the high production of caspase-1, IL-18, and IL-1β for the activation of the NLRP3 inflammasome induced by Amyloid Beta (Aβ) in microglia during the positive feedback loop, which is produced during systemic neuroinflammation [123,124,125]. Interestingly, a decrease in the Aβ level and an improvement in the memory impairment of APP/PS1/NLRP3 -/- mice is related to a knockout of NLRP3 [126]. Therefore, the NLRP3 inflammasome has gained attention since its relevance in neurodegenerative and neuroinflammatory diseases gained importance in new research [127,128,129].

### 2.4. Janus Kinase/Signal Transducer and Activator of Transcription (JAK/STAT): An Evergreen and Unconventional Pathway in Neuroinflammation and Neurological Dysfunctions

The proliferation and activity of cells present in the brain, such as neurons and astrocytes, are regulated by the JAK/STAT pathway. Neural Stem Cell (NSC) growth does not stop during brain development and can be observed in neurogenic areas, including the olfactory bulb’s Subventricular Zone (SVZ). The JAK/STAT pathway regulates NSC growth so that cytokines, especially IL-15, are expressed by adult NSC in the SVZ, stimulating the action of STAT1, STAT3, and STAT5. Interestingly, blocking JAK prevents NSC multiplication [130].

In this scenario, NDs are also strictly linked to the JAK/STAT pathway. Alzheimer’s disease is related to a dysfunction in the JAK2/STAT3 pathway, in which nicotinic acetylcholine receptors activate this pathway and lower the Aβ neurotoxicity [131,132,133,134]. The pathogenesis of Alzheimer’s disease was also related to the inactivation of STAT3, in which its activated/phosphorylated configuration (p-STAT3) had a considerable decline in the hippocampal neurons of patients with the pathology compared to the control group [135]. Studies have shown that in mice, high amounts of Aβ intensify tyrosine phosphorylation and the transcriptional functioning of Tyrosine Kinase 2 (TYK2), which is related to high phosphorylation of STAT3 in brains with Alzheimer’s disease. Furthermore, when inhibited, JAK/STAT3 signaling blocks microglia and astrocytes’ activation in animal neurodegeneration prototypes [136]. The results of Porro et al. demonstrated that inactivation of the JAK/STAT pathway interrupted the amount of secretion of pro-inflammatory cytokines, promoting a rise in the levels of IL-4 and IL-10, causing a change in the protective phenotype and attenuating the inflammation present in neurodegeneration [137].

Through the activation of the JAK/STAT pathway, the polarization of pro-inflammatory macrophages becomes possible, having an intrinsic relationship with Interferon-Gamma (IFN-γ), so that a deficiency in this interferon, presented by mice, demonstrated a reduction in the amount of neuron loss present in the substantia nigra through the induction of 1-Methyl-4-Phenyl-1,2,3,6-Tetrahydropyridine (MPTP), which is also related to the Parkinson’s Disease [138]. In their study, Qin et al. [139] demonstrated the relationship between the JAK/STAT pathway in microglial activation, its vital importance in the recruitment of cytokines and chemokines, and its function in Parkinson’s disease. The research investigated a possible therapy using a JAK/STAT pathway blockade through the JAK1 and JAK2 inhibitor AZD1480. The action of α-SYN activated the JAK/STAT pathway in macrophages and microglia, and the role of AZD1480 was to block the Major Histocompatibility Complex Class II (MHC-II), which is induced by α-SYN and, consequently, decreases the activation of STAT1 and STAT3 due to inflammatory gene inhibition in macrophages and microglia. The results showed that therapy with AZD1480 promoted an interruption of neuroinflammation stimulated by α-SYN, preventing microglia activation, the passage of CD4+ T cells and macrophages, and the formation of pro-inflammatory cytokines and chemokines. Notably, in vivo, the JAK/STAT pathway disruption prevented the deterioration of dopaminergic neurons. Therefore, Qin et al. demonstrated the relationship between neuroinflammation, neurodegeneration, and JAK/STAT pathway suppression. In this context, inhibition of the already activated JAK/STAT pathway may be a positive alternative for treating Parkinson’s disease [140].

## 3. Neuroinflammation and Microglial Activation: Charting the Path Forward for Alzheimer’s Disease, Parkinson’s Disease, and Multiple Sclerosis

It is crucial to understand that between 5 and 10% of cells in the CNS are microglia, and these have different morphologies closely related to their function and the local environment, such as amoeboid, medusa, rod, and branched [141]. It is essential to understand that in a scenario of damage or injury, microglia begin to have an amoeboid morphology with larger cell bodies and smaller branches [142,143]. Furthermore, microglial cells work with the blood-brain barrier (BBB) to defend the brain against damage and attack pathogens [144].

When installed in the brain, self-renewal is how microglia maintain themselves, requiring the action of transcription factors, such as interferon regulatory factor 8 and PU.1, and cytokines, including Colony-Stimulating Factor (CSF)-1 and IL-34 [145]. For microglia to develop and be preserved, some components are essential, namely, Extracellular Signal-regulated Kinases (ERKs), which allow the multiplication and maintenance of microglia, and the Colony-Stimulating Factor 1 Receptor (CSF1-R), which configures as a tyrosine kinase receptor capable of propagating intracellular signals that have a central role in microglial multiplication, and CSF-1 activates it and IL-34 [146,147]. It is essential to point out that accelerated microglia depletion occurs after a CSF-1R blockade by pharmacological means [142]. What differentiates microglial cells from macrophages in the adult brain is the fact that microglia are capable of expressing Purinergic Receptor P2Y12 (P2RY12)/Purinergic Receptor P2Y13 (P2RY13), Transmembrane Protein 119 (TMEM119), and CD11B, with a low expression of CD45, while circulating macrophages reveal a high expression of CD45 and CD11B [148]. By expressing more than 100 sensory genes, microglia can establish a link with their microenvironment. Among these genes, we can mention complementing system receptors, which help them interpret these signals and act as necessary, in addition to TLR2, P2RY12, and Sialic Acid-Binding Ig-like Lectin H (SiglecH) [149].

There are some mechanisms for regulating microglial activity that play a fundamental role and deserve to be highlighted. PRRs are related to regulating innate immunity and recognizing PAMPs and DAMPs. Cytokine receptors are associated with the production of several cytokines, such as Transforming Growth Factor-Beta (TGF-β), TNF-α, and interleukins, since, in the brain, microglia are the largest producer of cytokines. Chemokine Receptors are linked to G Proteins (GPCRs) called CCL1 receptors (CCR1), CCR2, CCR3, CCR4, CCR5, CCR6, CCR7, CXCR1, CXCR2, CXCR3, CXCR4, and CXCR5. Neurotransmitter receptors regulate interactivity between neurons and microglia through the recognition of ATP and its metabolites, and it includes cholinergic receptors, glutamate receptors, adrenergic receptors, purinoceptors, and cannabinoid receptors. There is also expression in the microglia of the Triggering Receptor Expressed on Myeloid Cells 2 (TREM2), which is a receptor present on the surface of cells that is part of the extensive family of Immunoglobulins (Igs) and is triggered through the recognition of pathogens by TLRs so that TREM2 establishes a relationship with the adapter DNAX-protein-12 activation (DAP12) present in the internal part of the cell. In addition to these receptors, others participate in this regulation, such as phosphatidylserine, scavenger, and Fc receptors [150,151,152,153].

Microglial cells are classified by a macrophage-derived system that can sometimes be simplistic: M1 (classical) and M2 (alternative) [154]. First, M1, which is activated by IFN-γ and LPS and has a pro-inflammatory and neurodegenerative profile, is related to the formation of nitric acid, proteases, and pro-inflammatory cytokines (IL-6, IL-1β, TNF-α, and IL-12) through the expression of Nicotinamide Adenine Dinucleotide Phosphate Hydrogen (NADPH) oxidase, which produces Inducible Nitric Oxide Synthase (iNOS), and through the action of the STAT1, which acts via JAK1/JAK2 signaling [155,156]. M2, with an anti-inflammatory and healing profile, has the expression of anti-inflammatory cytokines (IL-10), type 2 helper T cell (Th2) recruiting factor CC chemokine ligands, and others [157]. M2 microglia generate neurotrophic growth factors, such as Brain-Derived Neurotrophic Factor (BDNF) [158]. Interestingly, some microglia, as they synthesize pro-inflammatory and anti-inflammatory markers, have an intermediate phenotype. In this context, IL-4 and IL-13 stimulate M2a-type microglia, which incites CD206, arginase, and chitinase 3-like 3. On the other hand, the M2b phenotype is characterized as an intermediate state of microglia due to the secretion of restorative and inflammatory markers. Furthermore, M2c encourages tissue remodeling and repair once the inflammatory issue is resolved, forming CD206, TGF-β, and CD163 [159,160].

Also noteworthy is the ability of microglia to express CD11b, which is known as a “priming” marker, such as MHC-II [161], TMEM119, Sal-like protein 1 (SALL1), P2RY12, and P2RY13, involved in the immune response of the adult brain. Furthermore, it is interesting to note that microglia constantly investigate the CNS to have a relationship with processes such as protein clustering, phagocytosis, the secretion of soluble factors, and the refinement of the neural circuit. In CNS damage, microglia are activated in stages and are complex, responding through the action of signaling molecules, such as chemokines and cytokines, to repair tissue [151,152,162].

It is essential to understand that to protect the vigilant phenotype of microglia and regulate their activity, the CD200-CD200R and CX3CL1-CX3CR1 axes are vital. However, as a result of aging, there is a reduction in communicability through these axes, a fact that is related to the decrease in the expression of CX3CR1 and the neural ligands CX3CL1 and CD200, creating a pro-inflammatory scenario [149,163,164]. Following this line of reasoning, in the brain of an older adult, chronic inflammation promotes immunosuppression that damages the distribution pattern of microglia and causes a severe reduction in branching [164]. Interestingly, only in the white matter are the number of microglia positive for Mac-2, an indicator of subpopulations of microglia that perform phagocytosis, positively regulated, which does not occur in the gray matter. Therefore, certain elderly people called “Superagers,” have fewer microglia in the cortical white matter and, consequently, have better cognitive function [165].

Microglia have an intrinsic relationship with Alzheimer’s disease, as can be analyzed in numerous studies, including that by Sun et al. [166], who observed 1542 genes that were differentially expressed in Alzheimer’s disease, covering changes characteristic of the state of microglia and the Alzheimer’s disease phase, which relate to 12 microglial transcriptional states that have been observed in 443 individuals. By investigating post-mortem brain samples at an advanced age from 217 patients with Alzheimer’s disease and 226 individuals considered controls, the transcriptome of 152,459 unique microglial nuclei was analyzed in six brain areas. The first step was to collect 174,420 immune cells to perform in silico brain screening of Single-Nucleus RNA Sequencing (snRNA-seq) datasets using previously known marker genes (STAR Methods), which formed 16 clusters. Among these, 12 clusters were microglia (0–8 and 10,012). CD74, CSF-1R, and C3 marked them, so they were determined as different microglial states based on their molecular signatures and functionalities. The homeostatic microglia (MG0) and its intense expression of homeostatic markers CX3CR1 and P2RY12 were subject to annotation and observation, as well as the neuron surveillance microglia (MG1), which has a fierce expression of neurotransmitter receptors, and MG3, which demonstrated significant improvement in ribosome biogenesis. Furthermore, three inflammatory states were described (MG2, MG8, and MG10), with an intense expression of both cytokines and cytokine receptors (IL-1B, IL-4R, IL-17R, IL-15, IL-10RA, and CCL3), which are closely related to immune responses, highlighting signaling through the action of cytokines, and the structuring of the inflammatory reaction. From this, the study analyzed 423 individuals considered controls, with early and late Alzheimer’s disease in the prefrontal cortex, evaluating the statistical significance of the divergences in cellular fractions. It was demonstrated that in Alzheimer’s disease, MG4 and MG8 presented fractions with a considerable increase, unlike MG1, which presented a decrease in the fraction. Furthermore, using Ionized Calcium-Binding Adaptor Molecule 1(Iba1) pan-macrophage markers used in immunohistochemistry, it was possible to observe that individuals with Alzheimer’s disease have nuclei that express P2RY12 with comparatively smaller fractions. With the analysis of the increased expression of Peroxisome Proliferator-Activated Receptor Gamma (PPARγ) evidenced in snRNA-seq data, an increased amount was observed in the fraction of PPARγ^+^P2RY12^+^ cells in Alzheimer’s disease patients. Linked to this is an enrichment of Forkhead Box P1 (FOXP1)^+^ Leucine-Rich Repeat Kinase 2 (LRRK2)^+^ microglia in this group, demonstrating an MG8 inflammatory state. The discoveries made through in situ hybridization confirm that inflammatory states and lipid processing states establish a correlation with diseases.

In Parkinson’s disease, dopaminergic neurons present in the Substantia Nigra Pars Compacta (SNpc) degenerate gradually and for a long time, and the filamentous protein α-SYN makes up the protein inclusions that are called Lewy bodies [167,168]. It is essential to understand that α-SYN can be presented as β-sheet-containing fibrils and plays the secondary role of a seed element for the aggregation of α-SYN, with self-aggregation being a pathological function. In Parkinson’s disease, a decrease in α-SYN clearance is related to chaperone-mediated macroautophagy and autophagy, favoring the formation of α-SYN inclusions. In cases where neurons overexpress α-SYN, exosomes are released, which can provoke a premature reaction from microglia [169,170]. Furthermore, via the activation of inflammasomes, especially NLRP3, the secretion of pro-inflammatory cytokines, such as IL-18 and IL-1β, are stimulated by α-SYN aggregates, a fact that establishes a negative correlation with the development of Parkinson’s disease [171,172]. α-SYN, in physiological situations, is also responsible for regulating the storage of dopamine in synaptic vesicles and, consequently, establishes a relationship with the expression and functioning of the Vesicular Monoamine Transporter 2 (VMAT2) in neurons in the substantia nigra, corroborating its relationship with Parkinson’s disease. In the interaction between α-SYN aggregates and VMAT2, a decrease in dopamine vesicular storage space would lead to a deficiency in synaptic transmission and, as an effect, the death of neurons [172,173].

It is necessary to point out that an increase in the functioning of the NLRP3 inflammasome causes a deficit in microglial autophagy in Parkinson’s disease, leading to a change to a neurotoxic condition in microglia [174]. Yan et al. [175] explored the relationship between the microglial NLRP3 inflammasome, the PARKIN gene, and Parkinson’s disease. Through the neuronal expression of NLRP3, PARKIN polyubiquitination occurs and triggers neurodegeneration, so the objective of the research was to understand the function of PARKIN in the activation of NLRP3 using the overexpression of PARKIN in microglia following treatment with LPS. Three tests were performed on PARKIN knockout mice and wild-type mice to qualify motor activity: the open field, rotarod, and pole tests. It was then discovered that in LPS-induced mice, the decrease in PARKIN function caused an abundant assembly of microglial NLRP3, contributing to motor deficiency and a reduction in the number of dopaminergic neurons present in the substantia nigra. Therefore, the evidence suggests that regulating microglial NLRP3 activation by PARKIN via polyubiquitination ameliorates neurodegeneration in Parkinson’s disease.

Multiple Sclerosis is a complex disease of the CNS that is chronic, neurodegenerative, inflammatory, and autoimmune, which affects more than 2 million people worldwide [176,177]. Although the etiology of the pathology still seems uncertain, some conditions are related to a greater risk of developing Multiple Sclerosis, including biological sex, genetic constitution, and geographic factors [178]. Ren et al. [179] demonstrated the relationship between Quaking (Qki), a Ribonucleic Acid (RNA)-binding protein, and the control of phagocytosis carried out by microglia in the process of demyelination and remyelination. The results indicate an intensification in the neuroprotective activity of microglia in cases of CNS injuries and neurodegenerative pathologies, such as Multiple Sclerosis, a fact related to more significant phagocytosis by Qki. Therefore, the essential role of microglia in remyelination was confirmed [180]. Moreover, disruption of the BBB depends on microglial activation. This injury to the BBB is characterized as a recurrent and fundamental aspect of the progression of Multiple Sclerosis, demonstrated by a study by Yu et al. [181], further highlighting a possible manipulation of microglia to protect the BBB and ensure an improvement in inflammatory demyelination.

## 4. Exploring Medicinal Plants in Neuroinflammation: Comprehensive Insights on Effects, Dosage, Mechanisms, and Clinical Applications

The studies on various medicinal plants and their phytochemicals present a compelling overview of potential therapeutic agents against neuroinflammation. For instance, the research on *Cleistocalyx nervosum* var. paniala indicates that the extract significantly reduces pro-inflammatory markers, such as Cyclooxygenase-2 (COX-2) and iNOS, while inhibiting the activation of NF-κB and its downstream effects. The effective doses, ranging from 5 to 100 μg/mL, reveal its capability to modulate the inflammatory response in TNF-α- and LPS-stimulated BV-2 cells [58,59]. These findings underscore the potential of this plant as a source of new anti-inflammatory agents, particularly for NDs. However, future research should focus on in vivo models to corroborate these results and rigorous safety assessments to establish a comprehensive profile in clinical settings [182]. The role of the BBB in regulating neuroinflammation and its impact on the efficacy of medicinal plants is a critical but underexplored area. Understanding whether and how bioactive compounds in these plants can cross the BBB is essential to evaluating their potential for treating central nervous system disorders. Although preclinical findings provide valuable mechanistic insights, the absence of a detailed analysis of clinical trials or real-world applications limits the translational potential of these findings. Data from human studies, such as safety, efficacy, and practical implementation, must be considered to bridge the gap between experimental research and clinical practice.

The efficacy of *Curcuma longa* is well-documented in the context of neuroinflammation, mainly through the action of curcumin and its derivatives. The included studies demonstrate a marked decrease in inflammatory cytokines and a reduction in NF-κB activation in LPS-stimulated BV-2 cells. The doses tested ranged from 12.5 to 200 μg/mL, highlighting the potential for *Curcuma longa* as a natural alternative for treating neuroinflammation and oxidative stress-related disorders [60,61]. Despite its promise, the variability in effective dosing observed across studies indicates a need for more standardized protocols and clinical trials encompassing diverse populations to comprehensively evaluate its efficacy [183]. The substantial divergence in reported dosages, ranging from 10 to 200 μg/mL in vitro and 50 to 200 mg/kg in vivo, underscores the challenge of translating preclinical findings into clinical applications. Addressing this requires systematic dose-response studies and modeling to establish scalable and clinically relevant dosing protocols. The therapeutic efficacy of medicinal plants is highly influenced by variability in their bioactive compounds, which can arise from environmental factors such as soil quality, climate, and agricultural practices, as well as genetic differences among plant species or cultivars. Critically evaluating these variations is essential to standardize dosages and ensure the reproducibility of results in clinical applications. The use and efficacy of medicinal plants are often influenced by cultural practices and geographic factors, which can impact both the availability and the perception of these remedies. For instance, the legal status and cultural acceptance of *Cannabis sativa* vary significantly across regions, affecting research, accessibility, and its integration into therapeutic protocols. Understanding these cultural and geographic variations is essential for developing globally applicable guidelines for medicinal plant usage.

*Cannabis sativa* has garnered attention due to its diverse compounds, including cannabinoids that exhibit anti-inflammatory properties in LPS-stimulated BV-2 cells. The included study indicated significant reductions in pro-inflammatory cytokines while also increasing levels of endogenous cannabinoids, suggesting a dual role in inflammation modulation and pain management [62]. While these findings open avenues for novel treatments targeting neuroinflammation and chronic pain, the study’s limitations, such as sparse clinical evidence and the challenges of standardizing cannabis strains, necessitate further research that focuses on specific cannabinoid profiles and their therapeutic implications in clinical settings. Barbalace et al. [63] used *Cannabis* essential oils to mitigate neuroinflammation in BV-2 cells. Their results were interesting in modulating NF-κB, NLRP3, and other pro-inflammatory proteins among neuronal cells, and their results are promising since using natural oils may offer new avenues for administration than oral. Jiang et al. [64] explored the effects of phenylpropionamides (PHS) from *Cannabis sativa* against an animal model of Parkinson’s. The treatment demonstrated efficacy in counteracting α-SYN deposition via enhanced autophagy, and the commercialization of the plant before the foliage stage can be effective in countering legislative deviations associated with *Cannabis* usage as a narcotic. Borgonetti et al. [65] studied the effects of *Cannabis sativa* oils in mitigating neuroinflammation during traumatic injuries of the spinal cord. The results were positive, and this could be a significant strategy to counteract neuroinflammatory traumatic injuries without synthetic medications, therefore favoring a more cost-effective and reduced-side-effect treatment. Zhou et al. [66] demonstrated that *Cannabis sativa’s* effects on memory and learning damage, improving neuroinflammatory mediator levels, could be explored as candidate therapies against Alzheimer’s or other dementias. However, the study possesses limitations since it does not address the signaling pathways associated with *Cannabis*’s effects against neuroinflammation and brain defects in the animal model. Shin et al. [67] went beyond this. They demonstrated the effects of *Cannabis sativa*’s inflorescence against neuroinflammation in deep cervical lymph nodes and the dura mater and depression of an animal model. Their results are promising in evaluating the plant’s effects against neuroinflammation progression involving organs beyond the brain. Effectively, *Cannabis* reduced depressive-like behaviors and improved the pro-inflammatory cytokines profile of the animals. Mast cells were inhibited from degranulation. However, the signaling pathways involved in the anti-inflammatory and antidepressant effects of the plant remain unknown. Therefore, whether the effects were associated with NF-κB or PPARγ intervention was not fully explicated.

The study on *Dioscorea nipponica* also reveals a promising mechanism for regulating neuroinflammation through the modulation of iNOS and COX-2 expressions in both in vitro and in vivo models. The effective doses were from 10 to 100 μg/mL for in vitro studies and 60 mg/kg for in vivo applications [68]. Moreover, an increased expression of BDNF and phosphorylated Cyclic AMP Response Element-Binding Protein (pCREB) in animal models indicates potential cognitive benefits. This highlights the need for further research into the long-term effects and safety of *Dioscorea nipponica* and its primary compound, Dioscin, particularly regarding their impact on human mood disorders.

Research involving *Centipeda minima* has highlighted its complex phytochemical composition, demonstrating significant anti-inflammatory effects through inhibiting various pathways, including NF-κB and iNOS expression. Li et al. observed a reduction in inflammatory mediators in both in vitro and in vivo settings, reinforcing its potential to develop comprehensive anti-inflammatory therapies against neuroinflammation [69]. However, the variability in phytochemical compositions underscores the need for future studies that systematically evaluate the contributions of individual components, which would enhance the reproducibility and standardization of therapeutic applications.

*Atractylodis rhizoma* Alba has effectively suppressed inflammatory markers in LPS-stimulated BV-2 cells. The mechanisms involved include the significant inhibition of NF-κB and MAPK signaling pathways critical in the inflammatory response [70]. The promising results call for integrative approaches in clinical applications. Still, the lack of long-term studies means that further investigations are necessary to determine the potential side effects and interactions with other therapeutic agents.

The potential of *Vaccinium bracteatum* in modulating neuroinflammation is also noteworthy, with studies showing significant reductions in pro-inflammatory cytokines and oxidative stress markers. The effective doses ranged from 2.5 to 20 μg/mL, illustrating its promise as an anti-inflammatory and antioxidant agent [71]. However, variability across studies necessitates additional research to clarify the underlying mechanisms and confirm its efficacy across different biological models and clinical scenarios. *Lonicera japonica* also exhibits multifaceted anti-inflammatory properties, significantly reducing markers of inflammation in vitro [72]. However, the complex composition of this medicinal plant necessitates further exploration into which specific compounds contribute most significantly to its therapeutic effects. Future studies should aim to isolate these components, evaluate their respective contributions to enhancing anti-inflammatory responses, and determine the optimal dosages for effective therapeutic outcomes.

In Eom et al.’s study, *Bambusae caulis* demonstrated promising reductions in key inflammatory markers and oxidative stress levels. The observed alterations in Heme Oxygenase-1 (HO-1) and Nrf2 signaling pathways suggest that this plant may hold potential for developing new treatments for neuroinflammation [73]. However, the need for additional studies to identify specific active components and optimize therapeutic protocols remains a priority for future research.

*Zingiberis* rhizome demonstrates substantial anti-inflammatory effects in vitro on LPS-stimulated BV-2 cells. The administration of 1 or 10 μg/mL of ginger extract for 24 h resulted in a marked reduction of Nitric Oxide (NO) and Prostaglandin E2 (PGE2) production, alongside decreased expression of COX-2 Messenger Ribonucleic Acid (mRNA). Additionally, the treatment led to lower levels of pro-inflammatory cytokines and downregulated the phosphorylation of MAPK signaling molecules (ERK1/2, p38 MAPK, and c-Jun N-terminal kinase [JNK]). Notably, there was also a reduction in NF-κB nuclear translocation, highlighting its potential in modulating neuroinflammatory pathways [74]. Given these promising findings, *Zingiberis* rhizome may be a valuable candidate for developing neuroinflammation interventions. However, further research is critical to optimize dosing strategies and clarify the underlying mechanisms of action to realize its therapeutic potential.

Lastly, rice is commonly utilized worldwide for cooking. In this scenario, Subedi et al. [75] used genetically engineered resveratrol-enriched rice to counteract neuroinflammation in vitro. The results demonstrated positive alterations in the pro-inflammatory biomarkers and modulation of pro-inflammatory signaling pathways from enriched rice usage. Still, these results are auspicious due to rice’s ubiquity. If future clinical trials demonstrate the effectiveness of this product, its commercialization worldwide could be a reality, and preventing neuroinflammatory disorders and diseases would be easily amplified to the general public.

Overall, while these studies highlight the potential of medicinal plants in regulating neuroinflammation, further research efforts are essential to validate findings across different biological systems, establish optimal dosages, and explore long-term effects in clinical populations. Future research directions could significantly benefit from integrating various advanced methodologies. For instance, genetic studies can be employed to investigate the molecular pathways affected by phytochemicals found in these plants. By utilizing gene expression profiling and RNA sequencing techniques, researchers could elucidate how specific phytochemicals modulate inflammatory pathways at the genomic level, providing insights into their efficacy and mechanism of action.

Immunological assays are also crucial for comprehensively understanding the immune responses elicited by these medicinal plants. Flow cytometry and Enzyme-Linked Immunosorbent Assay (ELISA) could quantify cytokine levels and other immune markers in response to treatment with various plant extracts. These essays could help identify the most effective extracts and dosages that reduce neuroinflammation and enhance neuroprotection. Additionally, investigating the immunomodulatory effects of these plants on various immune cell types, including microglia and astrocytes, could reveal how they influence the neuroinflammatory milieu in the CNS.

Molecular docking studies based on plants’ phytochemical profiles could also provide valuable insights into their potential interactions with key molecular targets involved in neuroinflammation. Using computational modeling, researchers can predict how phytochemicals bind to receptors or the enzymes involved in inflammatory pathways. Such studies could facilitate the identification of promising compounds for further development and help optimize their structures to enhance efficacy and reduce potential side effects.

Moreover, exploring different extracts, such as aqueous, alcoholic, or supercritical fluid extracts, may reveal varying levels of bioactive compounds and their corresponding effects on neuroinflammation. Comparative studies on the efficacy of these extraction methods could uncover the most effective approaches to maximize the therapeutic potential of medicinal plants. Investigating the influence of specific extraction techniques on the stability and bioavailability of phytochemicals will also be necessary, as this could directly impact their clinical applicability.

Finally, integrating nanomedicine and nanocarriers into the research framework presents an exciting avenue for enhancing the delivery of medicinal plant extracts to treat neuroinflammation. Nanocarriers have shown great promise for improving the delivery of plant-based therapies to the CNS. For instance, curcumin-loaded nanoparticles have improved permeability across the blood-brain barrier, enhancing their efficacy against neuroinflammation. Similarly, cannabinoid nanocarriers have shown potential in targeting specific CNS regions to modulate inflammatory pathways. Integrating such examples would enrich discussions on their clinical applicability. Researchers could improve phytochemicals’ solubility, stability, and bioavailability by employing nanoparticles as carriers. Formulating nanocarriers targeting neuroinflammatory sites in the CNS could significantly enhance therapeutic outcomes while minimizing systemic side effects. Furthermore, studies exploring the synergistic effects of combining multiple phytochemicals within nanocarriers could lead to more effective multi-targeted therapies for neuroinflammation.

A multifaceted research approach combining genetic studies, immunological assays, molecular docking, extraction method evaluations, and nanomedicine strategies will advance our understanding of medicinal plants for neuroinflammation. Such efforts can lead to the development of optimized therapeutic protocols that not only leverage the unique properties of each plant but also ensure their safe and practical application in clinical settings. By addressing these areas, future research can provide a more comprehensive understanding of the role of medicinal plants in mitigating neuroinflammation, ultimately contributing to innovative treatment strategies for related neurological disorders.

## 5. Conclusions and Future Research Directions

**Figure 2:**
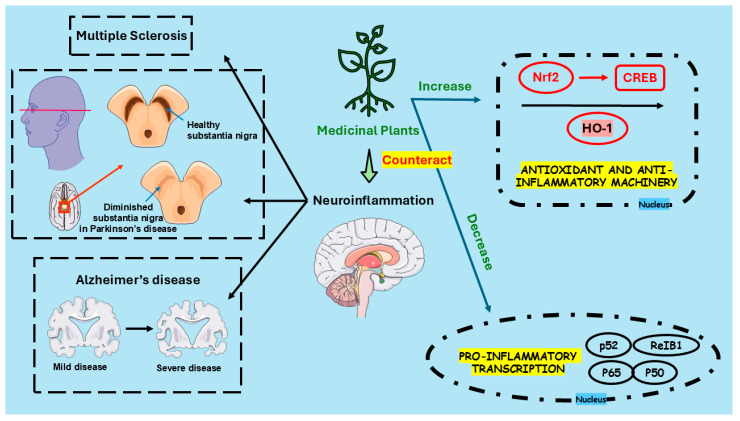
Key actions of medicinal plants in countering neuroinflammation and related disorders. Abbreviations: CREB, Cyclic AMP Response Element-Binding Protein; HO-1, Heme Oxygenase-1; Nrf2, Nuclear Factor Erythroid 2-Related Factor 2.

Exploring medicinal plants in neuroinflammation has yielded promising insights, revealing their potential as valuable therapeutic agents. Studies on *Cleistocalyx nervosum* var. paniala, *Curcuma longa*, and *Cannabis sativa* demonstrate their ability to modulate inflammatory pathways and provide symptomatic relief for neurodegenerative conditions. However, the included studies present several significant limitations, which must be listed to maintain the transparency of our findings. Firstly, it is worth noting that most of the findings lack sufficient background in animal trials, animal validations, and in-depth safety profiles for the included medicinal plants. The included studies also did not establish essential mechanistic details in some of the included studies, especially with the BBB analysis of the medicinal plants’ component crossing. The included studies reported a variety of effective doses in diverse populations, complicating standardization and reproducibility. In addition, discrepancies between the in vitro and in vivo findings occurred, reflecting inconsistent methodologies in the research. Also, each plant exhibits unique mechanisms of action, from suppressing pro-inflammatory markers to enhancing neuroprotective factors, underscoring their multifaceted therapeutic potential. Figure 2 illustrates the primary mechanisms by which medicinal plants affect neuroinflammation and associated neuroinflammatory disorders.

Despite these encouraging findings, challenges remain. Variability in effective doses, the need for standardized protocols, and the complexity of phytochemical compositions necessitate further research. Standardizing the phytochemical content in plant extracts remains a critical issue, as variability across studies can hinder reproducibility and the clinical translation of findings. Developing universally accepted protocols for extraction and quantification will be pivotal in addressing these inconsistencies and advancing therapeutic applications. Rigorous in vivo studies, comprehensive safety assessments, and standardized clinical trials are essential to validate these findings and establish these plants’ clinical efficacy and safety profiles. Despite the promising therapeutic potential of medicinal plants, their safety profiles, including possible adverse effects and toxicity at varying dosages, remain inadequately addressed. Comprehensive assessments of these factors are crucial to ensure their clinical translation and minimize risks associated with long-term use or interactions with conventional therapies.

Future research should integrate personalized medicine approaches, tailoring treatments based on individual genetic, environmental, and lifestyle factors to enhance therapeutic efficacy and minimize adverse effects. Emerging areas such as epigenetic regulation and gut-brain interactions offer promising insights into neuroinflammation mechanisms. Epigenetic modifications, including DNA methylation and histone acetylation, can influence the expression of key inflammatory genes. Similarly, the gut microbiome impacts neuroinflammation via the gut-brain axis, with microbial metabolites, such as short-chain fatty acids, playing a crucial role in modulating immune responses. Additionally, the principles of green health care—emphasizing sustainability, ecological balance, and the use of natural therapies—should guide the development and application of medicinal plant-based treatments. For example, plants known as adaptogens exhibit complex and nonspecific effects on human health but with an increasing ability to adapt, develop, and survive under stress conditions. This is of particular interest because they can turn nearly ubiquitous, and recent research has addressed these plants as anti-neuroinflammatory, affecting NF-κB signaling and pro-inflammatory cytokines production, including prostaglandins and leukotrienes, therefore, suppressing or delaying their onset NDs and fighting against depression, anxiety, stroke, and even CNS infections [184]. Employing advanced methodologies, including genetic studies to elucidate molecular pathways, immunological assays to evaluate immune responses, and molecular docking studies to predict interactions with key inflammatory targets, will further advance this field. Artificial intelligence (AI) presents innovative tools for drug discovery, particularly in identifying bioactive compounds within medicinal plants. Machine learning algorithms and neural networks can accelerate the identification of phytochemicals with therapeutic potential, optimizing the screening process for anti-inflammatory and neuroprotective agents. Optimizing extraction methods and exploring nanomedicine for improved delivery could also enhance the therapeutic potential of these plants. Comparing the cost-effectiveness and mechanisms of medicinal plants with synthetic drugs is vital for guiding healthcare decisions. Medicinal plants often offer sustainable and affordable alternatives with unique mechanisms, such as turmeric’s anti-inflammatory properties, which may complement or improve synthetic options. In conclusion, a holistic and multifaceted research approach incorporating personalized medicine and green healthcare principles is crucial for advancing our understanding of medicinal plants in neuroinflammation.

## Figures and Tables

**Table 1:** Medicinal plant effects on neuroinflammation: in vitro and in vivo models, effective dosages, molecular mechanisms, clinical implications, and study limitations.

Plant Species	Organ Harvested	Main Bioactive Compounds	Models	Effective Dose(s)/Treatment(s)	Mechanisms	Clinical Implications	Ref.
*Cleistocalyx nervosum* var. *paniala*	Berry seed	Ferulic acid, aurentiacin, brassitin, ellagic acid, alpinetin, and resveratrol	TNF-α-stimulated BV-2 cells in vitro	5, 10, or 25 μg/mL CNSE incubated for 24 h in vitro	↓ COX-2 activation, ↓ iNOS function, ↓ TNF-α, IL-6 and IL-1β mRNA expression, ↓ p38MAPK and ERK 1/2 phosphorylation, ↓ NF-κB activation, ↓ p65 and IκB phosphorylation, and↑ HO-1 induction in vitro	Potential for new anti-inflammatory agents targeting neurodegenerative diseases. Could pave the way for natural, multi-targeted treatments.	[58]
	Berry seed		LPS-stimulated BV-2 cells in vitro	1, 5, 10, 25, 50, or 100 μg/mL CNSE incubated for 24 h in vitro	↓ NO production, ↓ iNOS mRNA expression, ↓ TNF-α, IL-6, and IL-1β mRNA expression, ↓ MAPK phosphorylation, ↓ p-JNK, p-ERK 1/2 and p-p38 levels, and ↓ NF-κB activation in vitro	This could contribute to developing targeted anti-inflammatory therapies with fewer side effects.	[59]
*Curcuma longa*	Rhizome	Curcumin, demethoxycurcumin, and bisdemethoxycurcumin	LPS-stimulated BV-2 cells in vitro	12.5, 25, 50, 100, 150, or 200 μg/mL CLE incubated for 24 h in vitro	↓ NO production, ↓ PGE2 production, ↓ iNOS and COX-2 expression, ↓ TNF-α, IL-6 and IL-1β mRNA expression, ↓ NF-κB activation, ↓ IκB-α phosphorylation and degradation, ↓ p65 nuclear translocation, ↓ MAPK (p38, ERK, and JNK) phosphorylation, ↑ HO-1 expression, and ↑ Nrf2 nuclear translocation in vitro	It could enhance treatments for neuroinflammation and oxidative stress-related disorders, offering a natural alternative to synthetic drugs.	[60]
	Rhizome		LPS-stimulated BV-2 cells in vitro and scopolamine-induced male ICR mice in vivo	1, 10, 50, 100, or 150 μg/mL FCL incubated for 24 h in vitro and 50, 100, and 200 mg/kg FCL in vivo	↓ NO production, ↓ PGE2 production, ↓ iNOS and COX-2 expression, ↑ AChE inhibition in vitro, and ↑ pCREB and BDNF expression in vivo	It may offer new avenues for treating cognitive deficits and memory impairments associated with neurodegenerative conditions.	[61]
*Cannabis sativa*	Dried inflorescence	Cannabidiol, cannabigerol, cannabidiolic acid, tetrahydrocannabinol, β-caryophyllene, caryophyllene-oxide, α-humulene, apigenin, sesquiterpenes (E)-caryophyllene, N-trans-caffeoyloctopamine, N-trans-caffeoyltyramine, cannabisin A, cannabisin B, N-trans-coumaroyltyramine, N-trans-feryroyltyramine, cannabisin C, cannabisin D, cannabisin E, 3,3′-demethyl-grossamide, cannabisin M, isocannabisin N, cannabisin F, and grossamide	LPS-stimulated BV-2 cells in vitro	1 μg/mL CSE treated for four h in vitro	↓ TNF-α, IL-6, and IL-1β production, ↑ AEA and 2-AG expression, ↓ JNK and p38 activation, ↓ NF-κB nuclear translocation, and ↓ ROS production in vitro	It could be a cornerstone for novel treatments targeting neuroinflammation and chronic pain, with potential applications in psychiatric and neurological disorders.	[62]
	Dried inflorescence		LPS-stimulated BV-2 cells in vitro	5 × 10^−3^ μL/mL GG EO treated for two h in vitro	↓ iNOS, NLRP3, and COX-2 protein levels, ↓ NF-κB nuclear translocation, and modulated p38 MAPK and Akt in vitro	Using GG EO from essential oils could be an effective strategy against neuroinflammation targeting via venues other than oral. GG EO from commercial varieties may be attractive for the pharmaceutical industry.	[63]
	Seeds		1-methyl-4-phenyl-1,2,3,6-tetrahydro-pyridine-induced Parkinson’s disease model in male C57BL/6 J mice in vivo	350 or 700 mg/kg PHS from PHS-rich *Cannabis sativa* seeds in vivo	PHS enhanced autophagic biomarkers and improved α-SYN clearance in vivo	*Cannabis sativa* seeds can be a valuable strategy to counteract neuroinflammation in Parkinson’s. Cannabis seeds could be a strategy for commercializing the plant before the foliage stage.	[64]
	Dried inflorescence		LPS-stimulated BV-2 cells in vitro and spared nerve injury CD1 mice model in vivo	250 μg/mL treated for four h in vitro and 25 mg/kg cannabidiol-rich non-psychotropic *Cannabis sativa* in vivo	↓ Microglia pro-inflammatory phenotype through HDAC-1 and IκB-α inhibition and ↑ IL-10 expression in vitro and ↓ MAPK in vivo	*Cannabis sativa* could be a significant strategy to counteract neuroinflammation in traumatic neuronal injuries.	[65]
	Seeds		LPS-stimulated male Kunming mice in vivo	1 and 2 g/kg/day PHS from PHS-rich *Cannabis sativa* seeds in vivo	↓ IL-1β, IL-6, and TNF-α levels and ↓ hippocampal neuronal damage, and prevented learning and spatial memory damage in vivo	*Cannabis sativa*’s effects on memory and learning damages could be explored as candidate therapies against Alzheimer’s or other dementias.	[66]
	Dried inflorescence		LPS-stimulated male C57BL/6 mice in vivo	10, 20, and 30 mg/kg CSL 30 min before LPS administration in vivo	↓ Depressive-like behaviors, ↓ neutrophil-to-lymphocyte ratio, ↓ IL-1β and TNF-α, and inhibited mast cell degranulation in vivo	*Cannabis sativa* is effective in treating neuroinflammation beyond the brain and counteracting neuroinflammation in this central organ, preventing depression.	[67]
*Dioscorea nipponica*	Rhizome	Dioscin	LPS-stimulated BV-2 cells in vitro and scopolamine-induced male C57BL mice in vivo	10, 20, 50, or 100 μg/mL DNRE and Dioscin 200 and 400 ng/mL treated for two h in vitro and 60 mg/kg Dioscin in vivo	↓ iNOS and COX-2 expression, ↓ NO and PGE2 production, ↓ TNF-α, IL-6, and IL-1β mRNA expression, ↓ NF-κB nuclear translocation, ↓ IκB phosphorylation, ↓ p65 nuclear translocation in vitro, and ↑ BDNF and pCREB expression in vivo	It may support treatments to improve cognitive functions and mood disorders by targeting neuroinflammatory pathways.	[68]
*Centipeda minima*	Leaves	Chlorogenic acid, caffeic acid, rutin, isochlorogenic acid A, isochlorogenic acid B, isochlorogenic acid C, and 6-O-angeloylplenolin	LPS-stimulated BV-2 cells in vitro and LPS-stimulated male C57BL/6J mice in vivo	2, 4, or 6 μg/mL ECM incubated for 24 h in vitro and 100 and 200 mg/kg ECM in vivo	↓ NF-κB nuclear translocation, ↓ IκB phosphorylation, ↓ COX-2 and iNOS expression, ↓ PGE2 production, ↓ NOX proteins in vitro, ↓ PGE2, TNF-α, IL-6, and IL-1β production, ↓ NF-κB nuclear translocation, ↓ iNOS, COX-2, NOX2, and NOX4 expression in vivo	Potential to develop comprehensive anti-inflammatory therapies targeting multiple pathways involved in neuroinflammation.	[69]
*Atractylodis japonica* or *Atractylodes macrocephala*	Rhizome	Atractylenolide I, atractylenolide III, and atractylodin	LPS-stimulated BV-2 cells in vitro	10, 50, or 100 μg/mL ARAE incubated for 24 h in vitro	↓ NO production, ↓ TNF-α, IL-6, and IL-1β mRNA expression, ↓ iNOS and COX-2 expression, ↑ HO-1 mRNA expression, ↓ NF-κB activity, and ↓ MAPK, p38, ERK, and JNK activation in vitro	It may contribute to integrative approaches for treating neuroinflammation and related conditions.	[70]
*Vaccinium bracteatum*	Aboveground parts (not specified)	Quercetin, chrysin, apigenin, kaempferol, and lutelin	LPS-stimulated BV-2 cells in vitro	2.5, 5, 10, or 20 µg/mL VBME treated for 30 min in vitro	↓ NO and PGE2 production, ↓ iNOS and COX-2 expression, ↓ NF-κB p65 nuclear translocation, ↓ TNF-α, IL-6, and IL-1β levels, and ↓ ROS production in vitro	It may inspire new anti-inflammatory and antioxidant treatments with fewer side effects.	[71]
*Lonicera japonica*	Flower buds	Chlorogenic acid, caffeic acid, cryptochlorogenic acid, artichoke, isochlorogenic acid A, isochlorogenic acid B, isochlorogenic acid C, rutin, hibisin, and loganin	LPS-stimulated BV-2 cells in vitro	0.5, 5, 2.5, 5, or 10 µg/mL LJ treated for 30 min in vitro	↓ NO and PGE2 production, ↓ iNOS and COX-2 mRNA expression, ↓ TNF-α, IL-1β, MCP-1, and MMP-9 production, ↓ ROS levels, ↓ p38 MAPKs, ERK 1/2, JNK, and PI3K phosphorylation, ↓ JAK1/STAT1/3 phosphorylation, and ↓ NF-κB nuclear translocation in vitro	This could lead to new treatments targeting both neuroinflammation and related oxidative stress.	[72]
*Phyllostachys nigra* var. *henonis* or *Phyllostachys bambusoides*	Caulis	(-)-7′-epi-lyoniresinol 4,9′-di-O-β-D-glucopyranoside (7), (-)-lyoniresinol 4,9′-di-O-β-D-glucopyranoside (8), and bambulignan A	LPS-stimulated BV-2 cells and glutamate-stimulated hippocampal HT22 cells in vitro	10, 20, 40, 60, or 80 μg/mL BCE incubated for 24 h in vitro	↓ NO, TNF-α, IL-1β, and IL-6 levels, ↓ iNOS and COX-2 expression, ↓ ROS production, ↑ HO-1 mRNA expression, and ↑ Nrf2 nuclear translocation in vitro	Potential for advancing treatments against neuroinflammation and oxidative damage through modulation of key inflammatory and oxidative pathways.	[73]
*Zingiber officinale*	Rhizome	Gingerols and shogaol	LPS-stimulated BV-2 cells in vitro	1 or 10 μg/mL GHE incubated for 24 h in vitro	↓ NO and PGE2 production, ↓ COX-2 mRNA expression, ↓ TNF-α and IL-1β production, ↓ MAPK molecules, ERK1/2, p38 MAPK, and JNK phosphorylation, and ↓ NF-κB nuclear translocation in vitro	A promising candidate for developing interventions targeting neuroinflammatory processes and related molecular pathways.	[74]
*Oryza sativa* var. *japonica*	Seed	Resveratrol	LPS-stimulated BV-2 cells in vitro	1–100 μM RR incubated for 24 h in vitro	↓ NO, iNOS, and COX-2, MAPK modulation, and ↓ NF-κB translocation in vitro	Using RR could be a strategy to mitigate neuroinflammation widely since this could be a product of world commercialization.	[75]

Abbreviations: ↑, Increase; ↓, Decrease; 2-AG, 2-Arachidonoylglycerol; α-SYN, Alpha Synuclein; AChE, Acetylcholinesterase; AEA, Anandamide; Akt, Protein Kinase b; ARAE, *Atractylodis rhizoma* Alba Ethanolic Extract; BCE, Bambusae caulis in Taeniam Ethyl Acetate; BDNF, Brain-Derived Neurotrophic Factor; CSE, *Cannabis sativa* Extract; CSL, *Cannabis sativa* L. inflorescence extract; CLE, *Curcuma longa* Extract; CNSE, *Cleistocalyx nervosum* Ethanolic Seed Extract; COX-2, Cyclooxigenase 2; pCREB, cAMP Response Element-Binding protein; DNRE, *Dioscorea nipponica* Rhizome Ethanol Extract; ECM, Ethanol Extract of *C. Minima*; ERK, Extracellular Signal-Regulated Kinase; FCL, Fermented Curcuma Longa; GG EO, Gorilla Glue Essential Oil; GHE, Ginger Hexan Extract; HDAC-1, Histone Deacetylase 1; HO-1, Heme Oxygenase 1; IκB, Nuclear Factor of Kappa Light Polypeptide Gene Enhancer in B-Cells Inhibitor; IL-1β, Interleukin 1β; IL-10, Interleukin 10; IL-6, Interleukin 6; iNOS, Inducible Nitric Oxide Synthase; IκB-α, Nuclear Factor of Kappa Light Polypeptide Gene Enhancer in B-Cells Inhibitor Alpha; JAK/STAT, Janus Kinase/Signal Transducer and Activator of Transcription; JNK, C-Jun N-Terminal Kinase; LJ, *Lonicera japonica*; LPS, Lipopolysaccharide; MAPK, Mitogen-Activated Protein Kinase; MCP-1, Monocyte Chemoattractant Protein-1; MMP-9, Matrix Metalloproteinase 9; mRNA, Messenger Ribonucleic Acid; NLRP3, Nucleotide-Binding and Oligomerization Domain-Like Receptor Family Pyrin Domain-Containing 3; NO, Nitric Oxide; NOX, NADPH Oxidase; Nrf2, Nuclear Factor Erythroid 2-Related Factor 2; NF-κB, Nuclear Factor Kappa B; PGE2, Prostaglandin E2; PHS, Phenylpropionamides; PI3K, Phosphatidylinositol 3-Kinase; RR, Resveratrol-Enriched Rice; ROS, Reactive Oxygen Species; TNF-α, Tumor Necrosis Factor-Alpha; VBME, *Vaccinium bracteatum* Methanol Extract.

## Data Availability

No new data were created or analyzed in this study. Data sharing is not applicable to this article.
